# Supplementary material for: Cabozantinib Is Effective in Melanoma Brain Metastasis Cell Lines and Affects Key Signaling Pathways
Source: Int J Mol Sci. 2021 Nov 14;22(22):12296. doi: 10.3390/ijms222212296 (PMC8621572; doi:10.3390/ijms222212296)
Supplement: Supplementary file 1 [file ijms-22-12296-s001.zip › ijms-1444016-supplementary/ijms-1444016-supplementary.pdf]

Supplementary Figures

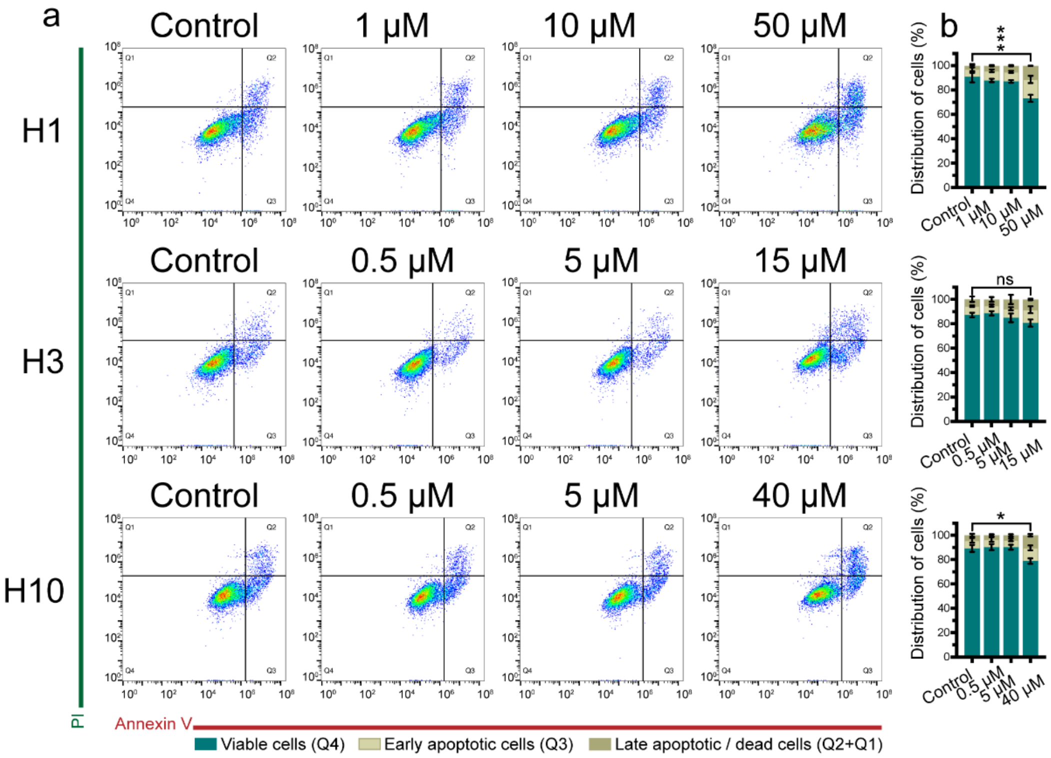

Supplementary Figure S1 Cabozantinib induces apoptosis in MBM cell lines. **(a)** Representative dot plots of cells untreated (control) or treated with different doses of cabozantinib as indicated for 24 h. Annexin V labels apoptotic cells, while propidium iodide (PI) labels necrotic cells. **(b)** Quantification of the percentage of viable, apoptotic and necrotic cells in H1, H3 and H10 cell cultures as untreated (control) or after 24 h exposure to cabozantinib. The experiments were done in triplicate. Abbreviations: Q1: necrotic cells, Q2: late apoptotic cells, Q3: early apoptotic cells, Q4: viable cells, ns: not significant, *: $p < 0.01$ in late apoptotic cells between control and cells treated with highest drug dose, ***: $p < 0.001$ in late apoptotic cells between control and cells treated with highest drug dose.

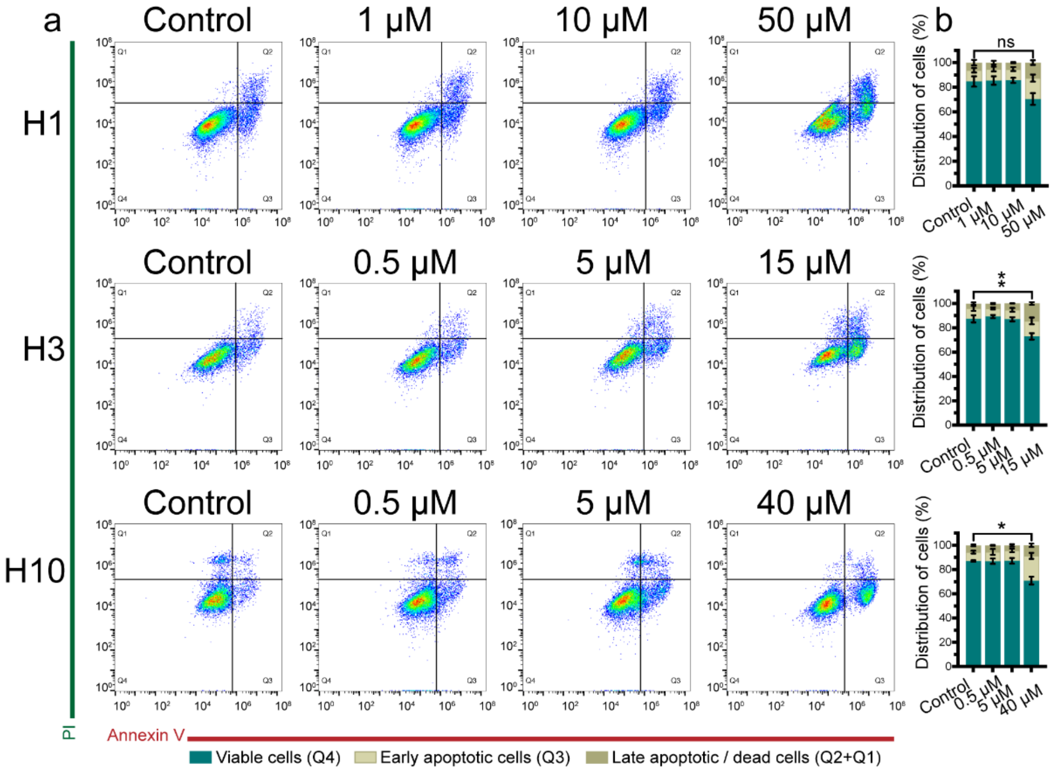

Supplementary Figure S2 Cabozantinib induces apoptosis in MBM cell lines. (**a**) Representative dot plots of cells untreated (control) or treated with different doses of cabozantinib as indicated for 48 h. Annexin V labels apoptotic cells, while propidium iodide (PI) labels necrotic cells. **(b)** Quantification of the percentage of viable, apoptotic and necrotic cells in H1, H3 and H10 cell cultures as untreated (control) or after 48 h exposure to cabozantinib. The experiments were done in triplicate. Abbreviations: Q1: necrotic cells, Q2: late apoptotic cells, Q3: early apoptotic cells, Q4: viable cells, ns: not significant, *: $p < 0.01$ in early apoptotic cells between control and cells treated with highest drug dose. **: $p < 0.01$ in late apoptotic cells between control and cells treated with highest drug dose.

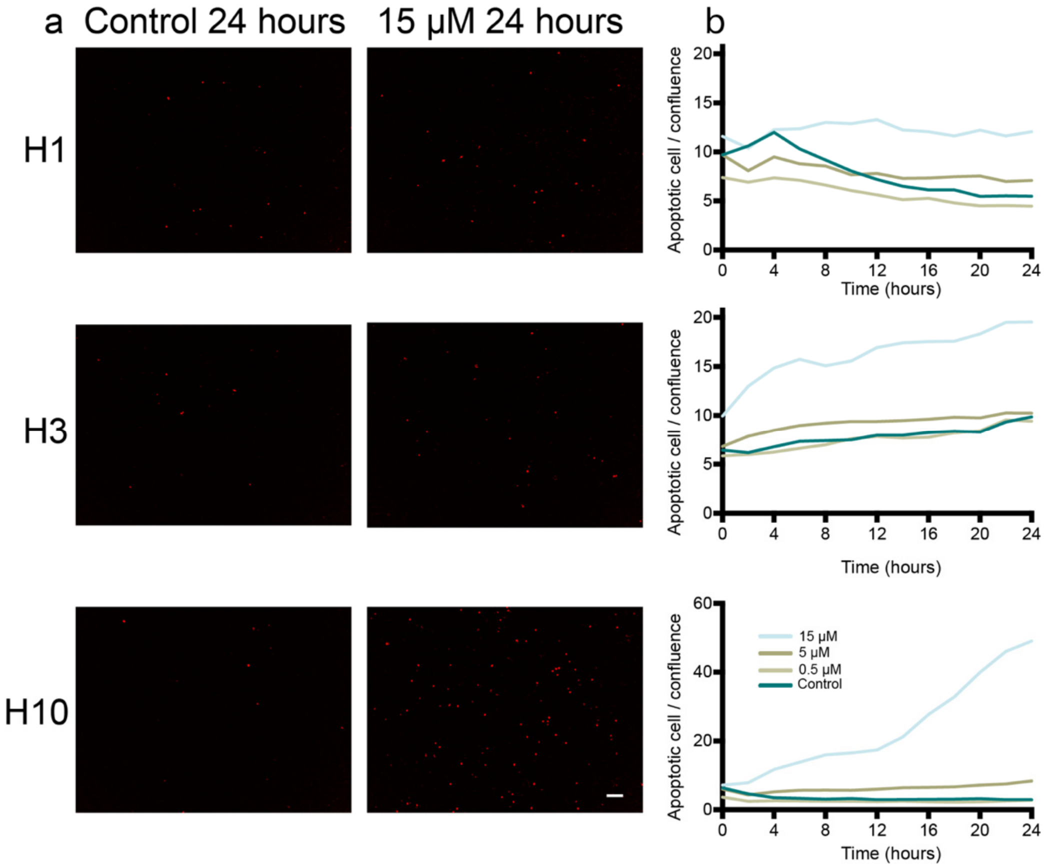

Supplementary Figure S3 Cabozantinib induces cleavage of caspase 3/7 in MBM cell lines. **(a)** Representative images of H1, H3 and H10 cells treated with 0.5 μM, 5 μM or 15 μM cabozantinib compared to untreated cells (control). Caspase 3/7 positive cells are shown in red. **(b)** Graphic representation of changes in caspase 3/7 levels during 24 h of exposure to 0.5 μM, 5 μM or 15 μM cabozantinib, compared to untreated cells. Scale bar = 100 μm.

p-RTK	p-value
p-IGF-1R*	0.0001
p-Insulin R	0.0003
p-EphA10	0.0047
p-Tie-2	0.006
p-MerTK*	0.0095
p-RYK	0.0099
p-EphA6	0.0213
p-DDR1*	0.0265
p-VEGF R2	0.0296
p-EphB6	0.0308
p-EphB1	0.0365
p-ALK	0.0415
p-EphA4	0.0457
p-EphA7	0.0484
p-PDGF Rb	0.0653
p-PDGF Ra*	0.0662
p-EphB2	0.0691
p-MSP R	0.0735
p-EGFR	0.0755
p-EphA1	0.0835
p-VEGF R3	0.089
p-ErbB4	0.0894
p-TrkC	0.1053
p-ROR1	0.1064
p-FGF R4	0.125
p-FGF R1	0.1484
p-EphB4	0.1533
p-TrkA	0.1689
p-VEGF R1	0.1963
p-ErbB3	0.2229
p-Flt-3	0.2265
p-HGF R	0.2449
p-ErbB2	0.2594
p-TrkB	0.2638
p-ROR2	0.264
p-SCF R	0.2764
p-MuSK	0.2885
p-Axl	0.3309
p-EphA5	0.3396
p-FGF R2a	0.353
p-c-Ret	0.5426
p-Dtk	0.5488
p-EphB3	0.554
p-Tie-1	0.6754
p-EphA3	0.7144
p-DDR2	0.8188
p-FGF R3	0.9168
p-M-CSF R	0.9237
p-EphA2	0.9834

Supplementary Figure S4 The list of p-RTKs tested with cabozantinib in the p-RTK array sorted after significant downregulation of protein expression**.** Abbreviations: * the p-RTKs selected to be tested in WB.

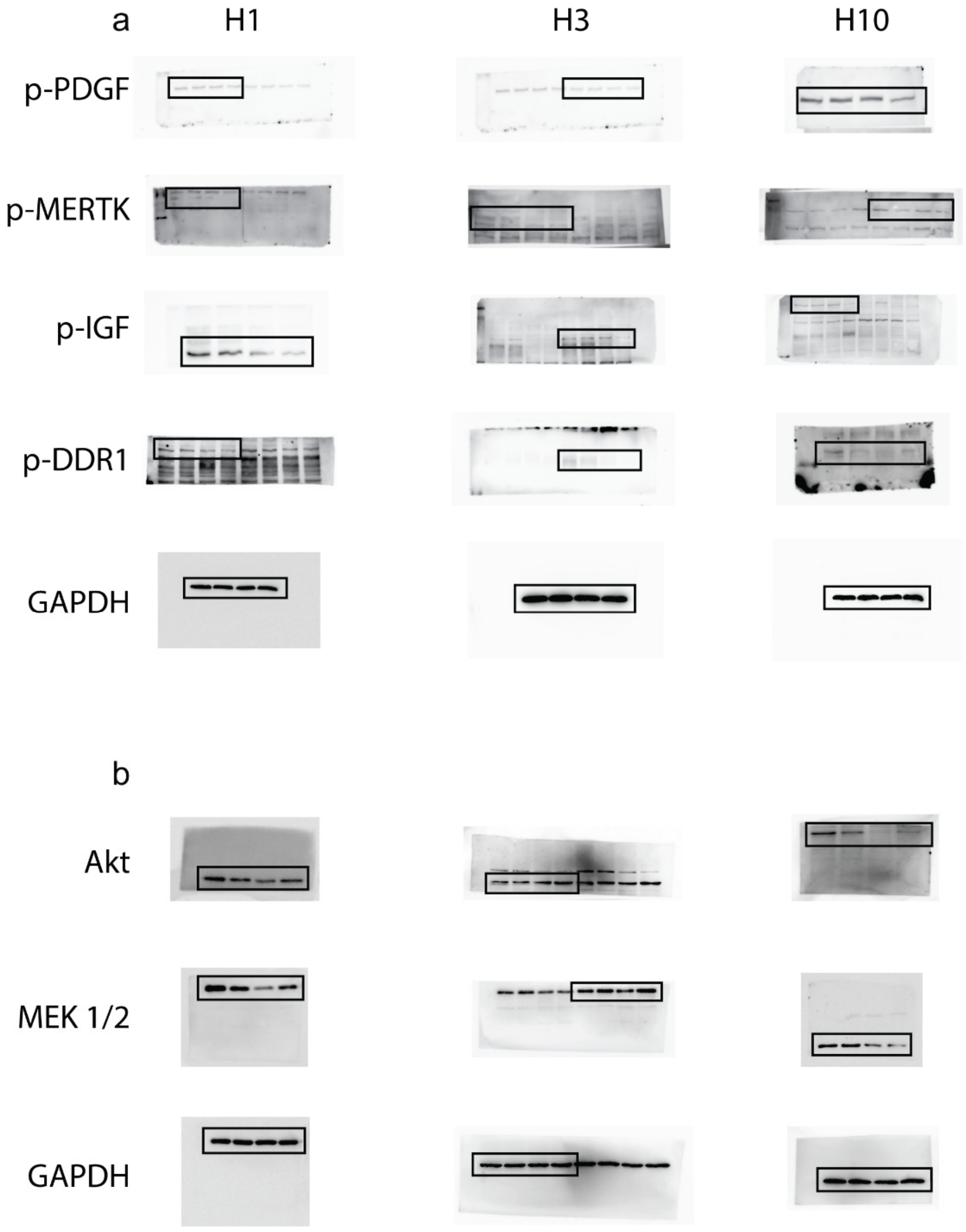

Supplementary Figure S5 Full-length blots. The frames indicate where the blots were cropped. Blots from Fig. 6 and 7 is represented by **(a)** and **(b)** respectively.
